# A COVID-19-Based Modified Epidemiological Model and Technological Approaches to Help Vulnerable Individuals Emerge from the Lockdown in the UK

**DOI:** 10.3390/s20174967

**Published:** 2020-09-02

**Authors:** Dario Ortega Anderez, Eiman Kanjo, Ganna Pogrebna, Omprakash Kaiwartya, Shane D. Johnson, John Alan Hunt

**Affiliations:** 1 School of Science and Technology, Nottingham Trent University, Nottingham NG11 8NS, UK; dario.ortegaanderez02@ntu.ac.uk (D.O.A.); Omprakash.kaiwartya@ntu.ac.uk (O.K.); 2 Business School, The University of Sydney, Abercrombie Building H70, Darlington, NSW 2006, Australia; gpogrebna@turing.ac.uk; 3 Alan Turing Institute, 96 Euston Road, London NW1 2DB, UK; 4 Jill Dando Institute, University College London (UCL), 35 Tavistock Square, London WC1H 9EZ, UK; shane.johnson@ucl.ac.uk; 5 Medical Technologies Innovation Facility, Nottingham Trent University, Nottingham NG11 8NS, UK; john.hunt@ntu.ac.uk; 6 College of Biomedical Engineering, China Medical University, Taichung 40402, Taiwan

**Keywords:** COVID-19, coronavirus, infection spread modelling, epidemiological model, contact tracing, personal protective equipment

## Abstract

COVID-19 has shown a relatively low case fatality rate in young healthy individuals, with the majority of this group being asymptomatic or having mild symptoms. However, the severity of the disease among the elderly as well as in individuals with underlying health conditions has caused significant mortality rates worldwide. Understanding this variance amongst different sectors of society and modelling this will enable the different levels of risk to be determined to enable strategies to be applied to different groups. Long-established compartmental epidemiological models like SIR and SEIR do not account for the variability encountered in the severity of the SARS-CoV-2 disease across different population groups. The objective of this study is to investigate how a reduction in the exposure of vulnerable individuals to COVID-19 can minimise the number of deaths caused by the disease, using the UK as a case study. To overcome the limitation of long-established compartmental epidemiological models, it is proposed that a modified model, namely SEIR-v, through which the population is separated into two groups regarding their vulnerability to SARS-CoV-2 is applied. This enables the analysis of the spread of the epidemic when different contention measures are applied to different groups in society regarding their vulnerability to the disease. A Monte Carlo simulation (100,000 runs) along the proposed SEIR-v model is used to study the number of deaths which could be avoided as a function of the decrease in the exposure of vulnerable individuals to the disease. The results indicate a large number of deaths could be avoided by a slight realistic decrease in the exposure of vulnerable groups to the disease. The mean values across the simulations indicate 3681 and 7460 lives could be saved when such exposure is reduced by 10% and 20% respectively. From the encouraging results of the modelling a number of mechanisms are proposed to limit the exposure of vulnerable individuals to the disease. One option could be the provision of a wristband to vulnerable people and those without a smartphone and contact-tracing app, filling the gap created by systems relying on smartphone apps only. By combining very dense contact tracing data from smartphone apps and wristband signals with information about infection status and symptoms, vulnerable people can be protected and kept safer.

## 1. Introduction

Coronaviruses (CoV) are a large family of enveloped, positive-strand RNA viral diseases capable of infecting a variety of host species, including humans and several other vertebrates [1]. CoVs predominantly cause gastrointestinal and respiratory tract infections, inducing a wide range of clinical symptotic manifestations [2]. Before the latest strain of coronavirus SARS-CoV2 in December 2019, six human CoVs, including four endemic (HCoV-OC43, -229E, -NL63, and -HKU1) and two epidemic (SARS-CoV and MERS-CoV) viruses had been identified. Despite recent scientific progress and increasing levels of public hygiene worldwide, a combination of factors have led to the SARS-CoV-2 outbreak becoming a global pandemic with a real risk to life. Even though drastic containment measures have been applied internationally, as of 25 June 2020, over 9.5 million cases of COVID-19 have been reported worldwide, causing death to over 480,000 people [3].

While COVID-19 has the potential to infect every individual in the world, the disease has been particularly dangerous and exhibited a greater risk of mortality for those over 70 years of age or with underlying health conditions (e.g., high blood pressure; respiratory problems, etc.). Many countries, particularly developed nations, have increasingly aging populations. For example, in the UK, estimates from the Office for National Statistics [4] suggest that in 2018, 18.3% of the population were aged 65 or over. Add to this the number of younger individuals with underlying health conditions, and this suggests that approximately 20% of the UK population has a considerably high risk of death if infected. Clearly, understanding and modelling such risk factors is important as this can inform estimates of likely mortality rates as well as informing the type and targeting of prevention and control strategies.

Motivated by the disparate mortality rates across different groups in society, this work proposes a modified compartmental epidemiological model (SEIR-v) to study the impact of reducing the contact rate of vulnerable individuals on the potential to reduce the number of fatalities caused by the disease. The motivation behind the model was that traditional epidemiological models like SIR [5] and SEIR [6] assume equal contact and death rates for every individual in the population. That is, these are abstract models that ignore the significant individual, behavioral, and spatial variation that is observed in the population of any country. In contrast, SEIR-v provides a means of studying the progression of the number of fatalities when differing contention measures are applied to different groups of individuals, according to their vulnerability to the disease, while also accounting for the characteristic variability seen in the case fatality rates across these groups. As outlined in [7], the original mathematical models used by the British government to inform policy in the UK did not account for vulnerable people. In contrast, SEIR-v provides the opportunity to distinguish between vulnerable groups and low-risk groups, allowing policy recommendations for each segment, rather than applying a one-size-fits-all approach.

Predictions made with SEIR-v demonstrated the importance of minimising the chances of vulnerable people contracting the disease, with an estimated reduction of 3681 and 7406 further deaths if their exposure to the virus was decreased by only 10% and 20% respectively. However, the widespread policies of physical distancing implemented to reduce risk, may now have contributed to another health problem: loneliness. According to recent correspondence published in The Lancet [8], those who do not have close family or friends, and rely on the support of voluntary services or social care, may feel vulnerable. The impact on physical health, arising from mental health problems due to loneliness should not be underestimated and ignored. There is therefore a need to help vulnerable people exit the lockdown whilst addressing their continued protection, providing them with (for example) the means to participate in the contact-tracing process.

Here, we consider practical options for facilitating exit strategies from lockdown for vulnerable groups. In line with this, we provide a set of recommendations:

1. The use of wearable devices (henceforth, wearables) to enable vulnerable people to take part in contact tracing,
2. The development of effective incentive mechanisms to motivate people to engage in contact tracing,
3. The use of digital tools to maintain physical distancing and monitor health symptoms,
4. The use of personal protective equipment.

The most important measures of effectiveness are:

1. Reduced rates of transmission post-lockdown in vulnerable populations;
2. Fewer restrictions on the vulnerable post-lockdown with noticeable improvement in their well-being (many may already be suffering from loneliness and mental health problems due to the lockdown);
3. Ensuring that the vulnerable people and the hard-to-reach are connected and closely monitored.

## 2. Infectious Diseases Spread Modelling

**Figure 1:**
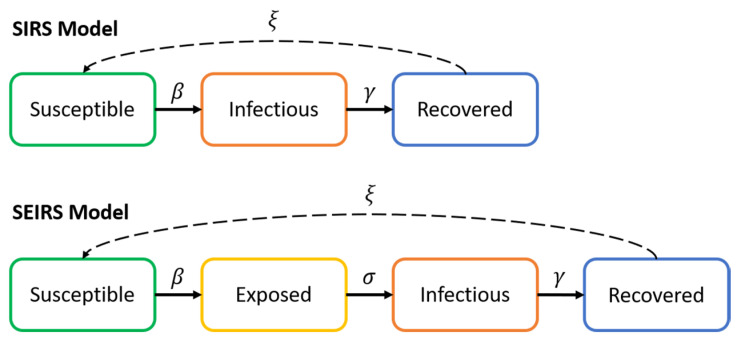
SIRS and SEIRS epidemiological models.

Generally, the spread of infectious diseases like COVID-19 are studied through the use of compartmental epidemiological models. Such models provide a simplified means of describing the transmission of the infectious disease through the different individuals within a population by dividing the individuals into different states regarding their current susceptibility to the disease and their disease transmission capabilities. Within such models, the SIRS (simplified version) [5] and the SEIRS [6] models are widely employed in the literature as simplified versions of the original model suggested by Kermack and McKendrick. A visual representation of the SIRS and SEIRS models is depicted in Figure 1. In addition to the SIRS and SEIRS models and based on the particular characteristics shown by COVID-19 in terms of the number of asymptomatic cases and the differing severity of the disease regarding various personal conditions, a number of extensions to SIRS and SEIRS have been established and studied which divide the population into sub-compartments based on age and other factors [9,10,11,12,13].

### 2.1. SIRS Model

The SIRS model divides the entire population into three different compartments, namely susceptible, infectious and recovered. The transition between one compartment to its following are controlled by the model transition parameters:

1. Infectious rate (*β*): is the rate of spread of the virus given by the probability of transmitting the disease between an infectious individual and a susceptible individual. This is subject to the disease transmission probability and the chance of contact.
2. Recovery rate $\gamma =\frac{1}{Tlat}$ is determined by the average duration of the infectious period of the disease (*T_lat_*).
3. Re-susceptibility rate (*ξ*) is the rate at which recovered individuals return to the susceptible state due to loss of immunity (normally ignored due to long-term immunity).

Given the definition of the above parameters, the SIR model can be expressed as per Equation (1):

(1) $$\begin{array}{c} \frac{dS}{dt}=-\frac{\beta IS}{N},\\ \frac{dI}{dt}\frac{dR}{dt}=\gamma I\\ \frac{\beta IS}{N}=-\gamma I, \end{array}$$

where *S*, *I* and *R* are the number of susceptible, infected and recovered (or removed) individuals respectively, and *N* is the total population, which follows *N* = *S*(*t*) + *I*(*t*) + *R*(*t*).

### 2.2. SEIRS Model

The SEIRS model proposed in [6] is a slight variation of the SIRS model, in that it includes an additional state ‘Exposed’ to the three states used in the SIRS model. The motivation behind this comes from the fact that some infectious diseases exhibit a considerable post-infection incubation period in which an infected person (exposed) is not yet infectious, thereby affecting significantly the dynamics of the transmission of the disease. The SEIRS model can be expressed as per Equation (2):

(2) $$\begin{array}{c} \frac{dS}{dt}=-\frac{\beta IS}{N},\\ \frac{dE}{dt}=\frac{\beta IS}{N}-\sigma E,\\ \frac{dI}{dt}=\sigma E-\gamma I\;,\\ \frac{dR}{dt}=\gamma I \end{array}$$

where *S*, *E*, *I* and *R* are the number of susceptible, exposed. infected and recovered (or removed) individuals respectively, and *N* is the total population, which follows *N =* *S(t) +* *E(t) +* *I(t) +* *R(t).* The parameter σ is given by $=\frac{1}{Tinc}$, where Tinc is the incubation period (the time it takes for an exposed individual to become infected).

### 2.3. The Role of Non-Pharmaceutical Interventions and Herd Immunity

Non-pharmaceutical interventions (NPIs) are those actions or measures employed with the aim of limiting the spread of a viral disease when pharmaceutical interventions, such as anti-viral medications and vaccines, are still not available. These are classified as either suppression or mitigation contention strategies depending upon whether the measures are intended to quickly reduce the reproduction number (the average number of secondary cases generated per typical infectious case), R, to values lower than 1, or to simply slow down the spread of the virus by controlling the value of R, while allowing it to take values in (R ≥ 1).

In other words, suppression strategies aim to turn the pandemic phase of the disease (R > 1) into the endemic phase (R < 1), where each infectious individual, on average, spreads the virus to less than one other person, thereby causing a decay in the daily number of new cases. In contrast, mitigation strategies, unless combined with certain levels of population immunity, are not aimed at the suppression of the virus per se. Instead, they are employed to reduce the health impact of the epidemic by controlling the curve through the contention of R, so that even though each individual, on average, spreads the disease to more than one person, such spread is to some extent controlled to meet the capacity of the respective health care system, while building up population immunity along the course of the epidemic phase. Ultimately, the accumulated immunity will prevent the disease from spreading any further, leading thereby to a rapid decline in the number of new infections and the consequent endemic phase of the disease.

**Figure 2:**
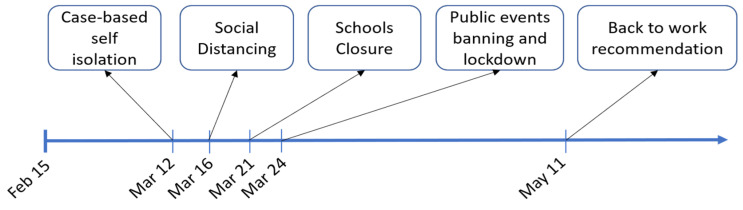
Non-pharmaceutical interventions applied by the UK government.

To date, hybrid strategies (e.g., see Figure 2) involving both mitigation and suppression measures, have been used to fight the spread of the epidemic in the vast majority of countries that have been severely affected by COVID19. Such strategies have included the implementation of diverse contention measures including instructing confirmed and suspected cases to self-isolate, encouraging social distancing, the banning of non-essential travel, mass gatherings and public events, the closure of schools and universities and the lockdown of the population. The latter has generally allowed for key workers to carry out their duties, and the general public (in lockdown at home) have been allowed to leave their homes for essential reasons including food shopping and exercise.

The analysis of existing data has demonstrated that mass testing and the isolation of infected individuals can on its own have a suppressive impact on the curve, significantly reducing the size of the peak [14]. Examples include the strategies followed by South Korea and Germany. However, it must be noted that the adoption of suppression strategies presents a challenge associated with the level of herd immunity achieved. Since this will by definition be low (if the strategy is effective), to avoid an increase of new infections, the contention measures have to be maintained until pharmaceutical interventions are available.

Although no empirical evidence exist to reject the possibility of reinfection from COVID-19, the results reported in studies that have investigated the persistence of antibodies in patients exposed to similar CoVs [15,16,17,18] suggest the antibody immunity built up by individuals exposed to the SARS-CoV-2 virus may potentially last until medical interventions are available. For instance, the study in [15] showed that antibodies in patients infected by the severe acute respiratory syndrome coronavirus (SARS-CoV), persisted for at least two years. Similarly, the results reported in [16] showed that 176 patients were found to maintain SARS-specific antibodies for 2 years. However, a significant reduction of immunoglobulin Gpositive was observed in the third year. Thus, SARS patients might be susceptible to reinfection 3 years after the initial exposure to the virus. Regarding the immunity to MERS-CoV, the work in [17] studied the antibody response in 9 healthcare workers in Jeddah, Saudi Arabia. The results outlined that antibody was detected for 18 or more months among those who showed symptoms of severe pneumonia. A further study in [18], explored the long-term antibody response against MERS-CoV. This research reported the persistence of antibodies in 6 out of the 7 (86%) explored individuals for at least 34 months after the outbreak.

## 3. Methods

### 3.1. Proposed Model: SEIR-v

**Figure 3:**
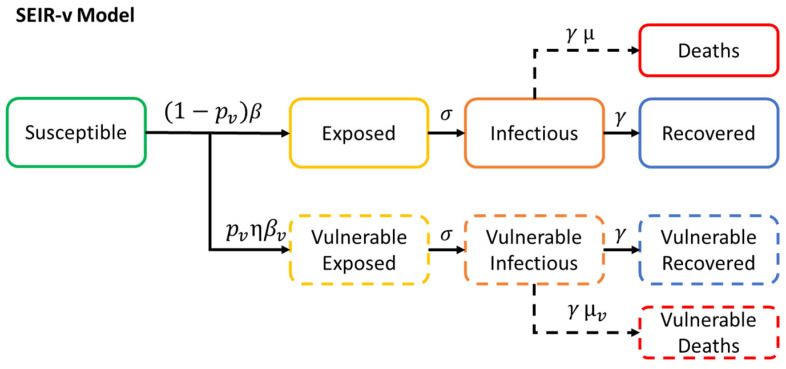
SEIR-v epidemiological model.

The need to develop the SEIR-v model comes from the consideration of the characteristic variability encountered in the severity of COVID-19 across different individuals with respect to their age group and state of health during the early phase of the infection. Evidence suggests, the mortality rate in individuals of advanced age and/or with underlying health conditions is significantly higher than that in younger healthier individuals [19]. Thereby, to inform strategies to reduce the number of fatalities, it is crucial to have a detailed means of looking more closely at the estimated impact of the application of the different NPIs across the different population groups with different profiles of vulnerability. SEIR-v provides a means of studying the impact of different NPIs on the number of deaths, when the former is applied only to those with higher vulnerability to the disease. The SEIR-v compartmental model can be defined as shown in Equation (3):

(3) $$\begin{array}{c} \frac{dS}{dt}=-\left(1-p_{v}\right)\left(I+I_{v}\right)\frac{\beta S}{N}-p_{v}\left(I+I_{v}\right)\frac{\eta \beta_{v}S}{N},\\ \frac{dE}{dt}=\left(1-p_{v}\right)\left(I+I_{v}\right)\frac{\beta S}{N}-\sigma E,\\ \frac{dE_{v}}{dt}=p_{v}\left(I+I_{v}\right)\frac{\eta \beta_{v}S}{N}-\sigma E_{v},\\ \frac{dI}{dt}=\sigma E-\gamma I\;,\\ \frac{dI_{v}}{dt}=\sigma E_{v}-\gamma I_{v}\;,\\ \frac{dR}{dt}=\gamma I\left(1-\mu \right),\\ \frac{dR_{v}}{dt}=\gamma I_{v}\left(1-\mu_{v}\right),\\ \frac{dD}{dt}=\gamma I\mu +\gamma I_{v}\mu_{v} \end{array}$$

where *S* is the number of susceptible individuals. *E*, *I*, *R* are the number of non-vulnerable exposed, infectious and recovered individuals respectively. *E_v_*_,_ *I_v_*, *R_v_* are the number of vulnerable exposed, infectious and recovered individuals respectively. *D* is the total number of deaths caused by the disease. *p_v_* is the probability of an individual being vulnerable to the disease. *β* and *β_v_* are the contact rates by non-vulnerable and by vulnerable individuals respectively. *σ* is the rate at which an exposed individual becomes infectious. *γ* is the rate at which an infectious individual recovers from the disease. *µ* and *µ_v_* are the case fatality rates for non-vulnerable and for vulnerable individuals respectively. However, it is clarified that two susceptible groups could be also used, having 80% of the population in S and the remaining 20% in $S_{v}$, in the model proposed, and then a similar outcome can be obtained with the use of the control parameter $p_{v}$ (probability of an individual being vulnerable to the disease). A conceptual diagram of SEIR-v can be seen in Figure 3.

### 3.2. Model Parametrization

Here, we present a model that is calibrated using data for the UK. Given that the disease is still spreading globally and key information is still unknown, definite values for the different parameters that define the spread of COVID-19 and the impact of the contention measures in place are still unknown. For instance, the number of asymptomatic cases and consequently the total number of individuals who are or have been infected by the disease remains to be determined. Consequently, the case fatality rate of the disease is also unknown. Motivated by this fact, first efforts were aimed at defining reasonable value ranges for the different model parameters through the comparison of the estimated number of deaths and the real number of deaths as reported in [20]. To do so, the incubation period (*σ*) and the recovery rate (*γ*) estimated in previous work were taken into consideration [21]. With these figures, the best fit parameters for the remaining variables were identified by running a Monte Carlo simulation with 100,000 iterations. With this, in each run the unknown parameters are allowed to take random values from a Gaussian distribution within their respective expected intervals. The definitions of the different model parameters can be found in Table 1. The differential equations provided in Equation (3) are then solved by substituting the different random and predefined parameters by their respective values.

### 3.3. Virus Transmissibility Study

Given the parameters provided in Table 1, the final mortality rate of COVID-19 was studied as a function of the percentage decrease in the contact rate of vulnerable individuals (*β_v_*). The mean values across the different Monte Carlo simulations and the reduction in the number of deaths caused by the disease estimated by the best fit model are provided in the Results Section.

## 4. Results

The results achieved by the analysis of the number of deaths caused by COVID-19 as a function of the contact rate of vulnerable individuals (*β_v_*) are presented in this section. Two different scenarios were considered:

1. The vulnerable group contact rate, *β_v_*, is decreased from the beginning of the outbreak. For this scenario, the potential reduction in the number of deaths if more protective measures for vulnerable groups had been applied from the beginning of the outbreak was studied.
2. The vulnerable group contact rate, *β_v_*, is decreased from June 2020. In this case. the potential reduction in the number of deaths resulting from the implementation of measures from June 2020 was studied.

It should be noted that the Fear Factor (*η*) already takes into consideration the widespread risk of the disease in vulnerable groups and the recommendation made by the UK government for vulnerable individuals to stay at home for at least 12 weeks at the beginning of the outbreak. The decrease in *β_v_* is therefore additional to that caused by *η*. Initial simulations using higher case fatality rates significantly overestimated the number of deaths caused by the disease in the UK as compared to those reported in [20]. It is thus believed the case fatality rate is significantly lower than that reported in [19].

### 4.1. Reduction of the Contact Rate of Vulnerable Individuals from the Beginning of the Outbreak

**Figure 4:**
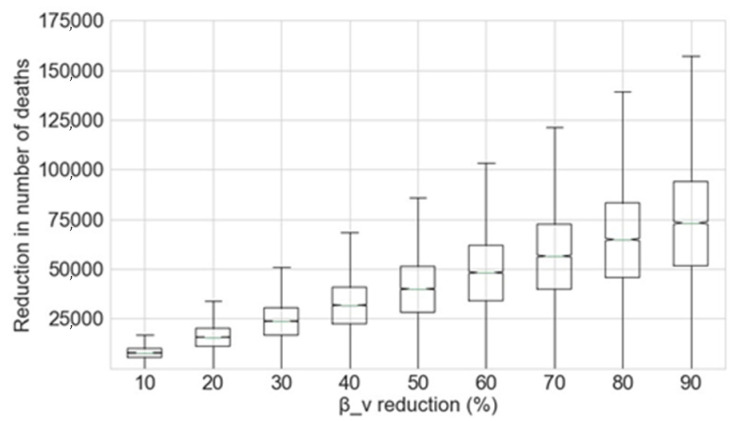
Reduction in the number of deaths as a function of the percentage decrease in *β_v_*, given that this reduction is applied at the beginning of the outbreak.

The potential reduction in the number of deaths caused by COVID-19 as a function of the percentage decrease applied to *β_v_* achieved by the Monte Carlo simulation is shown in Figure 4.

From this data, the reduction of the exposure of vulnerable groups to the disease at the beginning of the outbreak greatly decreases the number of deaths. The mean figures for the prevented deaths for each 10% decrease in *β_v_* from the beginning of the outbreak are shown in Table 2.

**Figure 5:**
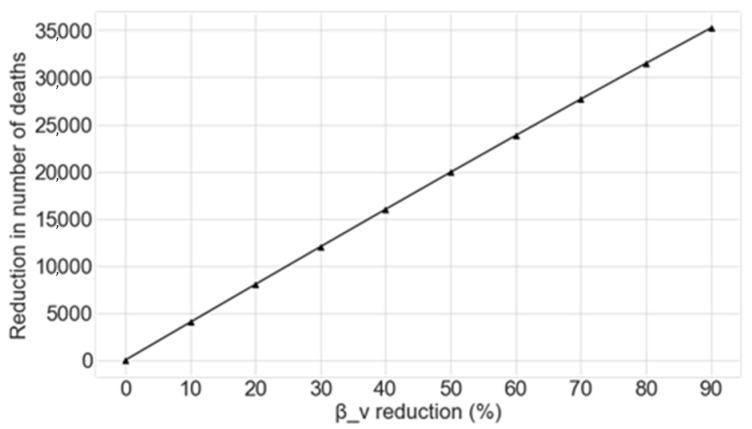
Reduction in the number of deaths as a function of the percentage decrease in *β_v_* for the best fit model, given that this reduction is applied at the beginning of the outbreak.

**Figure 6:**
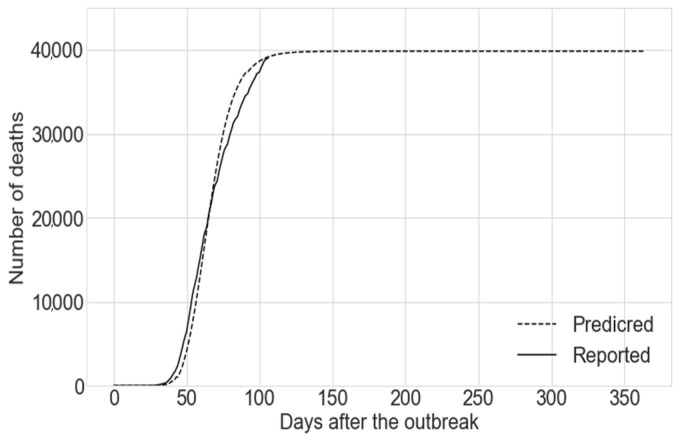
Predicted number of deaths using the best fit model vs. the number of deaths reported (real).

Similarly, the results obtained by the use of the best fit model across the Monte Carlo simulation are shown in Figure 5. The performance of this model at predicting the number of deaths caused by COVID-19 can be seen in Figure 6. While we propose a compartmental model which divides the population into different compartments, the main focus of the paper is to study the number of casualties caused by the disease and the potential lives which could be saved by reducing the exposure of vulnerable individuals to the disease. Given this, only the compartment “Deaths” is reported. The prediction given by the best fit model for the total number of deaths caused by COVID-19 in the UK at the end of the outbreak is 39,825. This figure is given by the compartment “Deaths” using the best fit model (the run exhibiting the smallest square Euclidean distance to the reported deaths up to the date when the simulation was run) amongst the different runs within the Monte Carlo simulation.

### 4.2. Reduction of the Contact Rate of Vulnerable Individuals from June 2020

**Figure 7:**
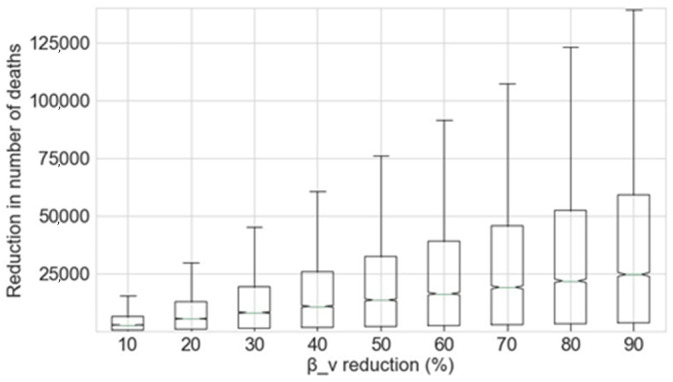
Reduction in the number of deaths as a function of the percentage decrease in *β_v_*, given that this reduction is applied from June 2020.

**Figure 8:**
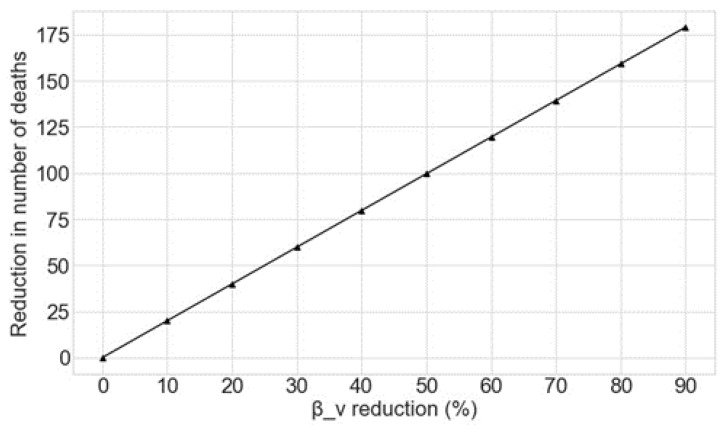
Reduction in the number of deaths as a function of the percentage decrease in *β_v_* for the best fit model, given that this reduction is applied from June 2020.

To compliment the estimates of the reduction in deaths associated with reducing the exposure of vulnerable people to the virus at the beginning of the SARS-CoV-2 outbreak, this section estimates the benefits of so doing from June 2020 onwards. The potential reduction in the number of deaths caused by COVID-19 as a function of changes to *β_v_* as estimated by the Monte Carlo simulation is shown in Figure 7. As can be seen, reductions in the exposure of vulnerable groups to the disease from June 2020 also greatly decreases the number of further deaths. The mean figures for the deaths prevented for each 10% decrease in *β_v_* from June 2020 are shown in Table 3. The results obtained by the use of the best fit model are shown in Figure 8.

### 4.3. Discussion

Despite the limitations of the model proposed, which assumes homogeneous mixing of the population, with no migration, births, or deaths from causes other than the epidemic itself, the results of the modelling suggest focusing the efforts on reducing the exposure of vulnerable individuals to COVID-19 can have substantial effects on the number of deaths caused by the disease. Motivated by this, three main points for consideration are proposed and discussed, namely (1) the use of dedicated wristbands for the vulnerable to enable social distancing and protect their well-being, (2) the use of Personal Protective Equipment (PPE) and (3) the relaxation of the lockdown easing policy and the benefits they can bring.

The purpose of next Section 5 is that of proposing ways of reducing the exposure of vulnerable individuals to the disease. While we justify these measures can have a positive impact towards achieving a reduction in the exposure, we are uncertain as to the exact impact those measures can have in reducing the exposure per se. Due to this, we studied the reduction in the number of deaths as function of the reduction in the exposure of vulnerable individuals to the disease by reducing the parameter $\beta_{v}$ from 10% to 90% in Figure 8.

## 5. Protecting the Vulnerable People

Contact tracing, which has been used alongside other protection measures across the world, can keep a record of any new infection cases and anyone who has been close to them [22]. This could enable uninfected and immune people to leave their homes, and people who might have been infected, could be instructed to self-isolate. However, contract tracing is time consuming and resource intensive and to be strictly accurate and valid would require 100% adoption. Bluetooth technology has been used previously for messaging and the tracking of nearby devices using proximity detection [23,24]. Apple and Google along with many health authorities have proposed smartphone hosted apps that use Bluetooth Low Energy (BLE) to automate the contact tracing process [25]. BLE is a form of wireless communication designed especially for short-range communication that is suitable for situations where battery life is preferred over high data transfer speeds. Unfortunately, vulnerable and older people are more likely to use older smartphones that are not equipped with the BLE feature, e.g., about 9–12% of smartphones in the UK lack the BLE functionality needed for it to work. In addition, data from Ofcom shows that while around 80 per cent of all adults owned a smart mobile phone in 2018, only 47% of 65–74 year old’s, and 26% of over 75s did [26]. This is supported by data published by Statista, which indicates that only 40% of over 65s used a smart phone to connect to the internet in 2019 [27]. Furthermore, many people have privacy concerns about using their smart phone as a tracer and might not be willing to download a contact tracing app. With this in mind, three possible solutions to support vulnerable people are proposed (1) contact Tracing wristband or wearable, (2) a social distancing alert mechanism and, (3) a wearable to monitor symptoms.

We want to highlight that in Section 5.5 we provide a number of mechanisms to incentivise the use of wearable technologies. It will bring a significant impact on the willingness of the people in wearing electronic devices. Also, in this area, a literature survey regarding this matter suggests that older adults are willing to adopt wearable technologies in case it has potential health and financial benefits in their life [28].

### 5.1. Wearables for Contact Tracing

**Figure 9:**
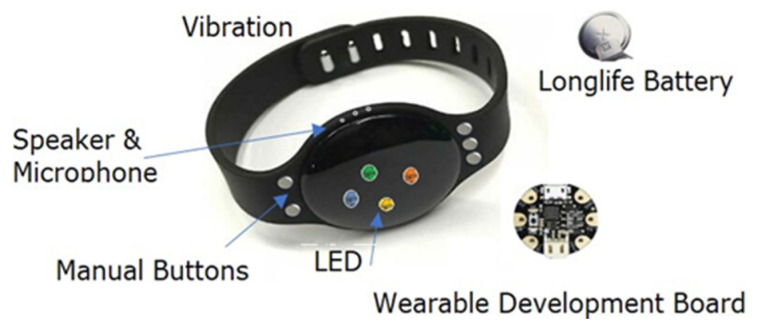
Wristband for vulnerable people.

The effectiveness of contact tracing hinges on how many people use it. It is proposed that governments could provide vulnerable individuals with a BLE wristband similar to the one in Figure 9, closing a data collection hole that would be created by systems relying on smartphones and an app only.

The wristband includes a proximity sensor powered by BLE. It also includes a manual control to self-report and change a wearers status, recording states like self-isolating, symptomatic and, tested negative or tested infected. When a user updates their state to indicate an infection after testing, this would update others they have been in close proximity with.

**Figure 10:**
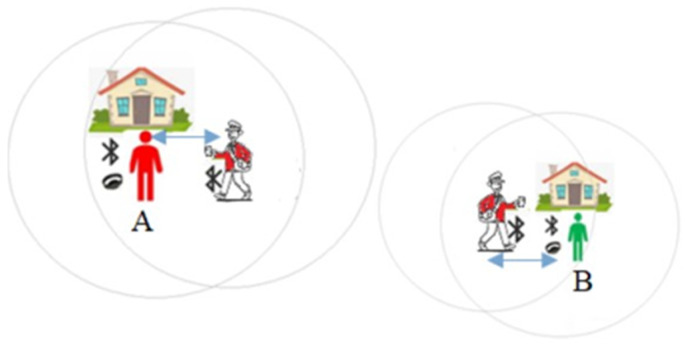
A schematic of wearable-based COVID-19 proximity tracing.

Figure 10 shows how the wristband concept works. Contacts of an individual wearing the wristband A are recorded on the wristband. A passer-by (e.g., postman) with a smartphone and Contact Tracing app comes within read range (longer range than close proximity) and downloads the records broadcasted by the wristband A. In turns. The individual wearing the wristband A could be alerted in real time using an LED light (or sound) of close proximity events.

We believe that adopting wearable (e.g., wristbands) solutions is advantageous over mobile apps for the following reasons:

1. Mobile phones might not be always with users. Instead, they might be left at home, in the car or at work, which means their social encounters don’t always correspond to actual contact.
2. A wristband solution will only have radio technology with a small memory and a battery with no access to users’ data which could help to preserve privacy. As it is low power it can always be on. It does not require set-up up or installation by the wearer.
3. Phone apps need to be installed and activated by users, and Bluetooth needs to be switched on. These requirements represent real barriers to the use of apps by vulnerable people who may have difficulties in remembering instructions, digital literacy, vision or motor control.
4. Contact Tracing apps can consume more energy as they are often kept active with battery optimisation features disabled.
5. Wearables are more likely to be worn at the front side of the body (e.g., wristbands, necklace or a keyfob), which could potentially improve the accuracy of the proximity detection in the case of face of face contact.
6. Smart phones come with different operating systems and settings, which means each model might require individual calibration and configuration.

A technical failure of any device is possible, the significance of that would depend on the quality of components, the design of the unit and the different parameters of providing a quality robust product. The wearable can be shipped with a long-life battery on the default settings. Beyond that ensuing that adoption and use can be supported or enforced by nations differently. The units are always on, they would not need any wearer interaction for the fundamental operation. Where a device went out into public spaces and failed this could be detected at read points. Like boarding public transport, entering public places where people gather. At these points, the device could be swapped and the wearer provided with replacement. For any devices to work, each individual in society needs to adopt the best practice, nations are already encouraging or enforcing that differently. The successful use of any tool and technology requires each person to use them, this type of approach facilitates that, without people requiring any skills or knowledge of the device in its simplest form. From which the value can be enhanced by wearer interaction.

### 5.2. Digital Tools to Maintain Social Distancing

By combining very dense contact tracing data from smartphone apps and wristbands signals with information about infection status and symptoms, vulnerable people can be protected and kept safe. Many countries have introduced wristbands for different purposes. For instance, in South Korea, people found to be violating lockdown rules can be ordered to wear a tracking band, which alerts the police if people leave the house [29]. The trackers were introduced after people started to leave their phones at home to avoid detection. The devices also alert the authorities if people try to remove it. Bulgaria has been testing Comarch LifeWristbands. developed in Poland [29]. In addition to confirming a person is staying at home, this system can monitor the wearer’s heart rate and be used to call the emergency services.

**Figure 11:**
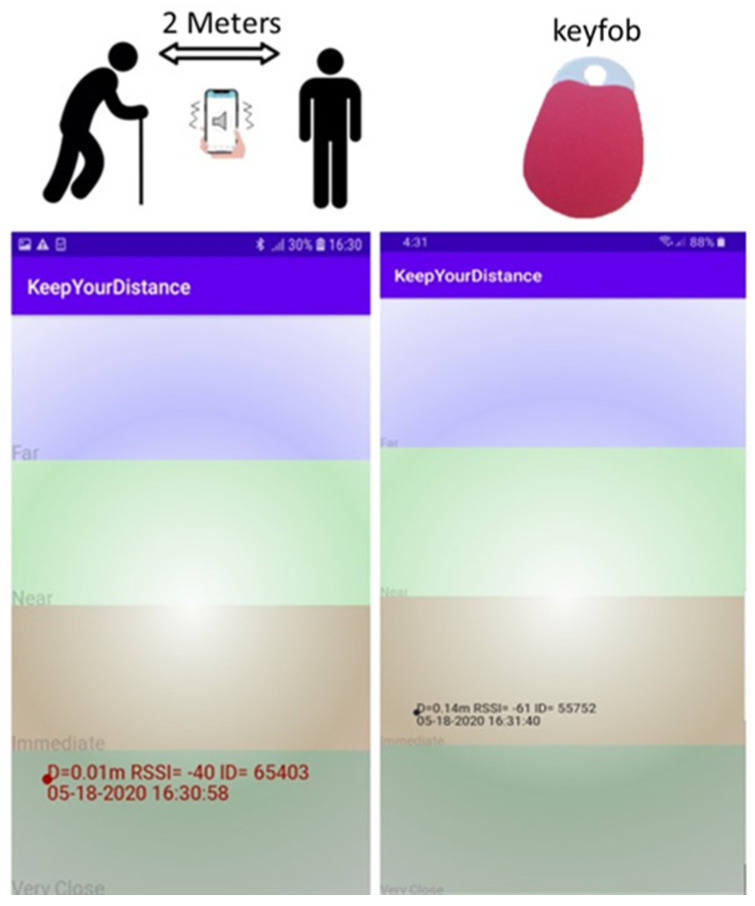
A BLE mobile app and a keyfob concept developed at Nottingham Trent University (NTU) to alert people when they are within 2 m proximity.

Contact Tracing apps and Internet of Things (IOT) devices such as key fobs, tags or wrist bands can also be used to alert people (e.g., using vibration) if another device comes within a specified distance. Figure 11 shows a BLE keyfob and KeepyourDistance app concepts being developed at Nottingham Trent University (NTU) to maintain social distancing. The KeepyourDistance screenshots show the signal strengths of nearby devices and the device vibrates when other phones with the same app are in close proximity.

### 5.3. Wearable to Monitor Symptoms

Dedicated health wristbands can be provided to vulnerable people with underling health conditions to track their health including: temperature, breathing and heart rate, and transmit it to their doctors. In addition to the above functionalities, most commercially available smart-wristbands and smart-watches incorporate an inertial measurement unit (IMU) composed of tri-axial accelerometers and gyroscopes. The work in [30] reports a face touching frequency of 23 times per hour with 44% of the face touches involving contact with a mucous membrane. Wristbands could be programmed to incorporate an alert mechanism to warn users whenever a potential movement of the hand towards the face is detected. The accuracy achieved by relevant gesture recognition work using inertial sensors [31,32] suggests that the development of such alerting features is feasible. These wearables can then be re-purposed once the COVID-19 epidemic is over. For example they can be given to older people and individuals with underling heath conditions to monitor their health and well-being. They might also be used to keep vulnerable people connected with local volunteers and community services.

### 5.4. Disease Transmission and the Use of Personal Protective Equipment

As outlined by the WHO [33], COVID-19 is primarily transmitted from person to person through small droplets exhaled by the mouth or nose when an infected individual speaks, sneezes or coughs. Direct transmission then occurs when such droplets travel onto the mucous membranes of susceptible recipients, necessitating contact at close range (usually within 1 m) [34]. In addition to this, exhaled droplets can also come to rest on surfaces around the infected individual. According to the WHO, the COVID-19 virus can survive up to 4 h on copper, 24 h on cardboard and up to 72 h on plastic and stainless steel surfaces. As a consequence, these become a potential source of infection when touched by susceptible individuals prior to touching their nose, mouth or eyes. Although exhaled droplets are often heavy enough not to travel great distances, sinking quickly to the ground or surrounding surfaces, viral particles in the form of bioaerosols can remain airborne for an extended period of time, particularly when droplet diameters are either too small for gravitational deposition (<2 µm) or too large for diffusive deposition (>200 µm) [35]. Experimental results on SARS during the 2003 epidemic [36] support these statements, showing viral particles in the form of bio-aerosols were being emitted by hospitalised patients. Likewise, recent work by [37] demonstrates aerosol COVID-19 remains viable and infectious with a half-life on the order of 1 h, thus confirming the plausibility of its transmission via airborne particles.

Given the above, the use of personal protective equipment (PPE) can be a key aspect to prevent the transmission of COVID-19. In this context, the use of masks and respirators can have a crucial role in safeguarding the mucous membranes and to prevent the transmission of viral droplets.

According to the European standards EN149:2001 and EN14683, there are four types of filtering masks, namely Filtering Face Piece 1 (FFP1), FFP2, FFP3 and surgical masks, with different models differing primarily in the filtration efficiency given by their capability to filtrate inwards and outwards particles. For the scope of this work, the use of surgical and FFP2 masks is discussed. In terms of inwards filtration, surgical masks are designed to protect against droplets, sprays and any other particle with a diameter greater than 100 µm [38]. FFP2 masks, however, retain >94% of the particles smaller than 0.5 µm. As the work in [39] indicated, exhaled particles can range from 0.01 to 1000 µm, with COVID-19 particles exhibiting a round or elliptic shape with diameters ranging from 0.06 to 0.14 µm [40]. As a consequence, surgical masks can prevent the inhalation of COVID-19 particles when these are expelled in the form of droplets with diameters greater than 100 µm but not when expelled in the form of small airborne particles. In contrast, FFP2 masks or respirators prevent the inhalation of both droplet and airborne viral particles. Despite the drawbacks encountered with surgical masks when it comes to the prevention of the filtration of small airborne particles, it must be noted that they can reduce the emission of viral particles into the environment [41]. It is worth noting that mask effectiveness decreases with increasing concentrations of water vapour and carbon dioxide between the face and the mask/respirator caused by each subsequent exhalation [38]. Thus, masks should be replaced frequently. It should also be noted that face masks to be fitted correctly and form a seal peripherally to stop air passing around the mask and not through it.

It should also be stated, the incorrect use of PPE, such as not changing disposable masks, can have a counterproductive effect, thus jeopardizing their protective effect and even increasing the risk of infection [38]. Given this, health organizations and government bodies should be disseminating accurate and clear information about how to wear and discard the different recommended protective equipment components properly.

### 5.5. Adoption and Incentive Mechanisms for Behavioural Change

**Figure 12:**
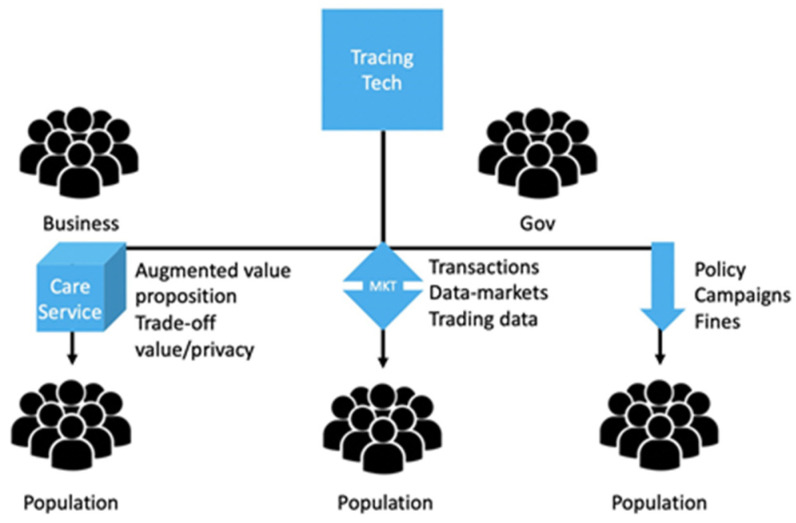
Adoption Approaches.

One of the challenges around implementing the proposed solutions is getting people to adopt and use them. In analogous settings, when young drivers were encouraged to use tracing technology solutions to improve their driving habits; young drivers, who participated in technology trials, only used behaviour monitoring apps while incentives were offered and stopped when the incentives stopped. Moreover, drivers often tried to “game” the system in order to obtain extra incentive points [42]. While the need to motivate individual adoption is universal, a segmented reaction to the proposed solutions and best practise is anticipated, so different strategies and incentive mechanisms might be required for different groups. Successful adoption depends on individual motivation, and incentives provided through a variety of strategies. The incentive mechanisms can range from providing information that is meant to resonate with basic values, such as using this technology will save lives to material and non-material incentives in the form of (i) paying people for their personal data (e.g., providing tax-rebates, etc.) or (ii) providing priority access to some services (e.g., medical services, etc.). For example, in the past, people have successfully adopted healthy behaviours when they received financial incentives [43]. From this, it follows that if incentives are offered people (a) adopt a certain behavior or (b) engage in a behavioral change. Rightly or wrongly these incentives may be more effective if they appeal to people’s ”present bias”, which is the tendency to pursue smaller and more immediate rewards (e.g., getting a small amount of money every day) rather than bigger and more abstract goals, such ”eradicating COVID-19 in the world”. Present bias is well documented not only in the behavioral science literature [44], but also in neuroscience [45], including the health context [46], and helps explain many behavioural regularities such as lack of altruism [47], lack of self-control [48], and even procrastination [49]. One potential incentivization mechanism could be offering a better value proposition to the user that goes beyond tracing. Considering that the vulnerable population generally tend to procure care services more often [50], the function of contact tracing is likely to succeed if it is embedded into the general care applications and services. An example of such general care services would be framing the contact tracing technology as a digital nurse for the vulnerable groups, that aims to monitor (in real time) the state of the wearer and potentially offer some desirable care features. Features such as health monitoring or being able to make automated calls for medical help in case the wearable detects signs of distress, for example [51]. Having a clear value proposition, which would go beyond the functional purpose of tracing would allow the technological solutions to succeed with the elderly population [52] more easily, as users will see not only how their data can benefit society, but also how their data can help them receive better, more efficient, and higher quality care. In such situations, due to the behavioural “privacy paradox” [53], users, who are concerned about privacy, are also likely to perceive the added value of the technological solutions as a trade-off between their personal data and desirable services. If the benefits of using the technology outweighs the cost (in terms of personal data loss), users will be much more likely to adopt the new wearables, even if that requires disclosing their location [54]. This, in turn, will significantly increase the chances of technology adoption as well as ensure policy success. Considering that people tend to attribute greater value and use products that have a cost associated with them (e.g., people see greater value in goods and services they need to pay for rather than free goods and services), such value proposition (i.e., offering medical or care service benefits in exchange for personal data) is likely to be self-sustainable. An alternative incentive mechanism can be implemented through material incentives by offering to buy personal data from the users (i.e., the users will receive financial remuneration for their personal data) [55]. This mechanism will leverage on participants’ willingness to trade privacy in the context of willingness to accept (WTA) money in exchange for data. The mechanism could work through offering a certain amount of money or an equivalent in discounts for users’ personal data in a clear and transparent transaction [56]. The key to success in this case is full transparency about (i) who is the data buyer (government or third-party organizations); (ii) how the data buyer will use the data; and (iii) how the data buyer will protect user data. The third strategy to push for adherence to the use of the proposed solutions, is through legal means. For example, contact tracing could be mandated by law and distributed to the eligible population. The use of the technology could then be monitored and “fines” for non-compliance could be introduced. The “fines” do not necessarily have to be monetary. They could be implemented as decreased benefits. The success of such this approach greatly depends on cultural values and social norms (in some societies punishment may work better than reward in policy implementations) [57]. The three suggested approaches are summarized on Figure 12. Two of the three proposed approaches involve monetary incentives, while one implies changes in care quality. Naturally, approaches which work with monetary incentives would require careful long-term planning in order to make sure that their effects are long-lived. The advancements in IoT and body area networking [58] are potential for data privacy [59] oriented smart tracing and tracking devices. 

## 6. Conclusions and Future Work

A key driver for this work was the large size of the vulnerable population and their higher risk of severe infection and death. This group is also prone to suffer from loneliness resulting from the prolonged period of lock-down. As such, governments need to balance the need to socially isolate vulnerable people and shield them while also taking into account their mental health and well-being which might also be severely affected by isolation measures.

A modified SEIR model, namely SEIR-v, through which the population was separated into two groups regarding their vulnerability to the disease was proposed to provide a means of studying the spread and the case fatality rate of COVID-19 when different contention measures are applied to different groups regarding that vulnerability. Using SEIR-v, the impact of a reduction in the exposure of vulnerable individuals to COVID-19 on the number of fatalities caused by the disease was analyzed. The results indicate an average of 3681 deaths can still be saved by only reducing by 10% the exposure of vulnerable groups to the disease from June 2020 onwards. In line with this, and also considering the negative consequences caused by the application of strict isolation measures on people’s mental health, a set of recommendations including the adoption of digital tools and protective equipment was proposed Future work will be directed towards the analysis of the lockdown easing steps taken by the U.K. government and their potential impact on further fatalities caused by COVID-19. The advancements in IoT and body area networking will be utilized to design and develop smart healthcare products for both wearable and indoor use cases to support the life after COVID-19.

## Figures and Tables

**Table 1:** Description of the SEIR-v model parameters.

Parameter	Unit	Description	Value	Comments
*N*	N People	Population	67,838,235 [15]	Total population in the UK as of 2020
*E_v_*_0_	N People	Vulnerable Exposed	2	Vulnerable individuals exposed to the disease at the beginning of the outbreak
*E*_0_	N People	Exposed	4	Non-vulnerable individuals exposed to the disease at the beginning of the outbreak
*I_v_*_0_	N People	Infected	0	Vulnerable infected individuals at the beginning of the outbreak
*I*_0_	N People	Infected	1	Non-vulnerable infected individuals at the beginning of the outbreak
*T_inc_*	Days	Incubation period	5.6 [16]	*σ =* 1/*T_inc_*, where *T_inc_* is the time it takes for an exposed individual to become infectious
*T_lat_*	Days	Latent period	7.5 [16]	*γ =* 1/*T_lat_*_,_ where *T_lat_* is the time it takes for an infectious individual to recover
*µ_v_*	Vulnerable deaths/Vulnerable Infected	Vulnerable Case Fatality Rate	[0.005–0.037, 95% CI]%	Case fatality rate of COVID-19 on vulnerable individuals
*µ*	Non-vulnerable deaths/non-vulnerable Infected	Non-vulnerable Case Fatality Rate	[0.000007–0.000011, 95% CI]%	Case fatality rate of COVID-19 on non-vulnerable individuals
*p_v_*	*-	Vulnerable probability	0.2	Probability of an individual being vulnerable to the disease
*η*	*-	Fear Factor	0.33	Fear factor caused by the recommendation made by the UK government for vulnerable individuals to stay at home for at least 12 weeks at the beginning of the outbreak and the widespread severity of the disease within this group
*β*_0_	1/(person*day)	Initial Contact Rate	[0.5–2.1, 95% CI]	Contact rate at the beginning of the outbreak
*β*_1_	1/(person*day)	Contact Rate 1	[0.9–0.95, 95% CI] * *β*_0_	Contact rate after the mandate of case-based self-isolation
*β*_2_	1/(person*day)	Contact Rate 2	[0.9–0.95, 95% CI] * *β*_1_	Contact rate after government encouragement for social distancing
*β*_3_	1/(person*day)	Contact Rate 3	[0.75–0.85, 95% CI] * *β*_2_	Contact rate after schools closure
*β*_4_	1/(person*day)	Contact Rate 4	[0.40–0.60, 95% CI] * *β*_3_	Contact rate after lockdown order and banning of public events
*β*_5_	1/(person*day)	Contact Rate 5	[1.1–1.9, 95% CI] * *β*_4_	Contact rate after recommendation for people to go back to work
*β_vi_*	1/(person*day)	Vulnerable Contact Rate	η * β_i_	Contact rate of vulnerable individuals.

*- Dimensionless.

**Table 2:** Relationship between the decrease in *β_v_* and the resultant number of deaths avoided when this decrease is applied from the beginning of the outbreak expressed as the mean value of the Monte Carlo simulation.

**Decrease in *β_v_***	10%	20%	30%	40%	50%	60%	70%	80%	90%
**Decrease in Number of Deaths**	7699	15,512	23,428	31,434	39,519	47,671	55,876	64,122	72,395

**Table 3:** Relationship between the decrease in *β_v_* and the resultant number of deaths avoided when this decrease is applied from June 2020 expressed as the mean value of the Monte Carlo simulation.

**Decrease in *β_v_***	10%	20%	30%	40%	50%	60%	70%	80%	90%
**Decrease in Number of Deaths**	3681	7406	11,172	14,975	18,810	22,673	26,559	30,464	34,383
